# Prognostic impact of liver fibrosis and steatosis by transient elastography for cardiovascular and mortality outcomes in individuals with nonalcoholic fatty liver disease and type 2 diabetes: the Rio de Janeiro Cohort Study

**DOI:** 10.1186/s12933-021-01388-2

**Published:** 2021-09-24

**Authors:** Claudia R. L. Cardoso, Cristiane A. Villela-Nogueira, Nathalie C. Leite, Gil F. Salles

**Affiliations:** Department of Internal Medicine, School of Medicine and Clementino Fraga Filho University Hospital, Universidade Federal do Rio de Janeiro, Rua Croton, 72, Jacarepagua, Rio de Janeiro, RJ CEP: 22750-240 Brazil

**Keywords:** Cardiovascular events, Cohort study, Liver fibrosis, Mortality, Nonalcoholic fatty liver disease, Steatosis, Transient elastography, Type 2 diabetes

## Abstract

**Background:**

Liver stiffness measurement (LSM, which reflects fibrosis) and controlled attenuation parameter (CAP, which reflects steatosis), two parameters derived from hepatic transient elastography (TE), have scarcely been evaluated as predictors of cardiovascular complications and mortality in individuals with type 2 diabetes and nonalcoholic fatty liver disease (NAFLD).

**Methods:**

Four hundred type 2 diabetic patients with NAFLD had TE examination (by Fibroscan^®^) performed at baseline. Multivariate Cox analyses evaluated the associations between TE parameters and the occurrence of cardiovascular events (CVEs) and mortality. TE parameters were assessed as continuous variables and dichotomized at low/high values reflecting advanced liver fibrosis (LSM > 9.6 kPa) and severe steatosis (CAP > 296 or > 330 dB/m). Improvements in risk discrimination were assessed by C-statistic and by the relative Integrated Discrimination Improvement (IDI) index.

**Results:**

During a median follow-up of 5.5 years, 85 patients died (40 from cardiovascular causes), and 69 had a CVE. As continuous variables, an increasing LSM was a risk marker for total CVEs (hazard ratio [HR]: 1.05; 95% CI: 1.01–1.08) and all-cause mortality (HR: 1.04; 95% CI: 1.01–1.07); whereas an increasing CAP was a protective factor for both outcomes (HR: 0.93; 95% CI: 0.89–0.98; and HR: 0.92; 95% CI: 0.88–0.97; respectively). As dichotomized variables, a high LSM remained a risk marker of adverse outcomes (with HRs ranging from 2.5 to 3.0) and a high CAP was protective (with HRs from 0.3 to 0.5). The subgroup of individuals with low-LSM/high-CAP had the lowest risks while the opposite subgroup with high-LSM/low-CAP had the highest risks. Both LSM and CAP improved risk discrimination, with increases in C-statistics up to 0.037 and IDIs up to 52%.

**Conclusions:**

Measured by hepatic TE, advanced liver fibrosis is a risk marker and severe steatosis is a protective factor for cardiovascular complications and mortality in individuals with type 2 diabetes and NAFLD.

## Background

Nonalcoholic fatty liver disease (NAFLD) is the most frequent chronic hepatic disease [1–3]. Diabetes is also a highly prevalent condition worldwide [4] and it is associated with a greater prevalence and severity of NAFLD [2, 3, 5]. There are complex interplays between diabetes and NAFLD [2, 5], and both are associated with higher risks of future development of atherosclerotic cardiovascular diseases (ASCVD) and excess mortality [6–8]. Indeed, a possible synergistic increase in cardiovascular risk of the association between diabetes and NAFLD has been suggested in a recent meta-analysis [9].

The presence of advanced liver fibrosis has been shown to be the most important prognostic marker of worse outcomes in patients with NAFLD [10, 11]. Liver stiffness measurement (LSM) by transient elastograghy (TE) is an accurate non-invasive method to identify fibrosis, especially severe advanced fibrosis and cirrhosis [12–14]. Similarly, the controlled attenuation parameter (CAP), also obtained by TE, is feasible to screen and quantify liver steatosis, but with lower sensitivity and specificity [14–16]. However, there are scarce longitudinal data that investigated whether LSM and CAP are associated with clinical outcomes in NAFLD [17–19], particularly in diabetic individuals [20].

Therefore, we aimed to investigate if LSM and CAP, assessed by TE performed at baseline, were associated with future adverse cardiovascular and mortality outcomes in a prospective observational cohort of individuals with type 2 diabetes and NAFLD.

## Methods

### Study overview

This is a longitudinal prospective study, nested within the Rio de Janeiro Type 2 Diabetes (Rio-T2D) cohort study, with 414 individuals with type 2 diabetes and NAFLD who performed hepatic transient elastography (TE) examination between 2012 and 2015 and were followed-up until December 2019 in the diabetes outpatient clinic of our University Hospital. All participants gave written informed consent, and the local Ethics Committee had previously approved the study protocol. The characteristics of the participants of the whole Rio-T2D cohort, as well as of the individuals with NAFLD, its baseline procedures and diagnostic definitions, all have been detailed previously [21–26]. All participants with NAFLD had at least a previous abdominal ultrasound demonstrating any grade of liver steatosis and none of them had any other cause of liver steatosis except NAFLD. Specifically, we excluded individuals with current weekly alcohol ingestion > 100 g, confirmed by patient and family members; with viral hepatitis, confirmed by serology; with other possible etiologies of liver disease, such as autoimmune or biliary obstruction; and using well-known steatogenic drugs, such as corticosteroids or methotrexate [21–23].

### Hepatic TE examination

All TE examinations were performed by a single experienced operator, blinded to clinical participants’ data, with the Fibroscan^®^ 502 equipment (Echosens, France), according to the instructions and training provided by the manufacturer [23]. All the measures were acquired with either the 3.5 MHz standard M probe or the 2.5 MHz standard XL probe (whenever there were unsuccessful measurements with the M probe). Measurements were performed on the right hepatic lobe through intercostal spaces with the patient lying in dorsal decubitus with the right arm in maximal abduction after at least a 2-h fasting. Ten successful acquisitions were performed on each patient. Both liver stiffness measurement (LSM, which indicates fibrosis) and controlled attenuation parameter (CAP, which indicates steatosis) were obtained simultaneously. The median value of the 10 acquisitions was the final LSM, expressed in kiloPascal (kPa) units, and the final CAP, expressed in decibels per meter (dB/m). The success rate was calculated as the number of successful measurements divided by the total number of measurements. Only procedures with at least ten valid measurements, a success rate of at least 60%, and an interquartile range (IQR)/median value of LSM ≤ 0.3 were considered reliable; otherwise the examination was discarded. Fourteen individuals (3.4%) had invalid TE examinations and were excluded from further analyses, totaling 400 individuals with type 2 diabetes and NAFLD with valid TE examinations in this study. The median success rate of the valid examinations was 91% (IQR: 83–100%), and the median IQR/median ratio of LSM was 0.14 (IQR: 0.1–0.19). According to cut-offs published in NAFLD patients, advanced liver fibrosis, corresponding to METAVIR stage ≥ F3, was defined by a LSM > 9.6 kPa [12]; and severe steatosis, corresponding to ≥ 66% liver steatosis, was defined by a CAP > 296 dB/m [27]. Alternatively, we also dichotomized CAP at a higher value (> 330 dB/m), as recently proposed to better define severe steatosis [14].

### Follow-up and outcomes

All participants were followed-up regularly at least 3–4 times a year until December 2019 under standardized treatment. The observation period for each patient was the number of months from the date of the TE examination to the date of the last clinical visit in 2019 or the date of the first endpoint, whichever came first. The primary outcomes were the occurrence of any cardiovascular event (CVE) and all-cause mortality. Total CVEs included the following: fatal or non-fatal myocardial infarctions (MIs), sudden cardiac deaths, new-onset heart failure, death from progressive heart failure, any myocardial revascularization procedure, fatal or non-fatal strokes, any aortic or lower limb revascularization procedure, any amputation above the ankle, and deaths from aortic or peripheral arterial disease [24–26]. Primary outcomes were ascertained from medical records, death certificates and interviews with attending physicians and patient families, by a standard questionnaire reviewed by two independent observers [24–26]. Secondary outcomes were the classic 3-point major adverse cardiovascular events (MACEs: non-fatal MIs and strokes plus all cardiovascular deaths), and cardiovascular and non-cardiovascular mortalities.

### Statistical analysis

Continuous data were described as means (SDs) or as medians (interquartile range [IQR]). Baseline characteristics of participants with high/low LSM and CAP were compared by independent t-tests, Mann–Whitney tests or by χ^2^ tests, when appropriate. Kaplan-Meier curves of cumulative endpoints incidence during follow-up, compared by log-rank tests, were used for assessing different incidences of outcomes between patients with high and low LSM and CAP. For assessing the prognostic value of the TE parameters (LSM and CAP) for each primary and secondary outcome, a time-to-event multivariable Cox analysis was undertaken, adjusted for the following potential confounders/risk factors: age, sex, body mass index (BMI), diabetes duration, smoking, arterial hypertension, presence of atherosclerotic cardiovascular diseases and microvascular complications at baseline, serum HbA_1c_, HDL- and LDL-cholesterol, and use of insulin, statins and aspirin. Both analyses with continuous and dichotomical (high/low) TE parameters were performed. Also, different analyses were performed with each TE parameter included separately and concomitantly into the same model. We also performed specific interaction and sensitivity analyses of subgroups according to selected clinical characteristics: age (≥/< 65 years), sex, BMI (obese/non-obese), smoking (never/past or current), and presence/absence of cardiovascular and microvascular complications. Finally, a separate analysis for the primary outcomes was carried out with individuals cross-classified into 4 subgroups according to high/low LSM and CAP values. These results were presented as hazard ratios (HRs) with their 95% confidence intervals (CIs). We also evaluated whether LSM, CAP or both were able to improve risk discrimination in relation to a basic standard risk factor model. For addressing it, we performed analyses using two metrics of model risk discrimination improvement: the increase in C-statistics (i.e., the area under curve applied to time-to-event Cox analysis) and the relative Integrated Discrimination Improvement (IDI) index, which measures the percentage increase in the discrimination slope after including the TE parameters in relation to that of the basic model [25, 26, 28]. In all analyses a two-tailed probability value < 0.05 was considered significant. Statistics were performed with SPSS version 19.0 (SPSS Inc, Chicago, IL, USA).

## Results

### Baseline characteristics

Mean LSM was 7.3 kPa (SD: 5.9; median: 5.8; IQR: 4.4–8.3 kPa), and mean CAP was 279 dB/m (SD: 59; median: 281; IQR: 237–322 dB/m). Overall, 59 individuals (15%) had a high LSM (> 9.6 kPa) indicating advanced fibrosis; and 163 (41%) had a CAP > 296 dB/m and 88 (22%) > 330 dB/m, indicating severe steatosis. LSM and CAP were moderately correlated (Spearman’s rho = 0.40). Table 1 shows the participants’ characteristics at TE performance and of those divided according to having or not advanced fibrosis and severe steatosis. Individuals with advanced fibrosis and with severe steatosis were younger, more obese, and had lower HDL-cholesterol levels than their counterparts without advanced fibrosis and severe steatosis, respectively. Moreover, patients with severe steatosis had a higher HbA_1c_ and serum triglycerides than those without severe steatosis. There were no differences between these subgroups regarding other cardiovascular risk factors, prevalence of cardiovascular and microvascular complications and diabetes treatment at baseline. Only 25 patients were using other antihyperglycemic drugs than metformin, sulphonylureas or insulin, and none of them were using GLP-1 agonists or SGLT-2 inhibitors.

**Table1:** Baseline characteristics and outcome incidences in all 400 patients evaluated and in those divided according to higher and lower liver stiffness measurement (LSM, indicative of liver fibrosis) and controlled attenuation parameter (CAP, indicative of liver steatosis) on transient elastography (TE) examination

Characteristics	All patients (n = 400)	Patients with LSM ≤ 9.6 kPa (n = 341)	Patients with LSM > 9.6 kPa (n = 59)	Patients with CAP ≤ 296 dB/m (n = 237)	Patients with CAP > 296 dB/m (n = 163)
Age (years)	64.4 (9.9)	64.7 (9.9)	62.6 (10.0)	65.5 (10.2)	62.9 (9.4)‡
Male sex, %	36.0	35.2	40.7	35.6	38.6
BMI (kg/m^2^)	30.4 (5.4)	29.9 (5.2)	32.9 (6.2)*	28.7 (5.1)	32.3 (5.1)*
Waist circumference (cm)	104 (12)	103 (11)	112 (12)*	100 (11)	109 (11)*
Smoking, current/past, %	43.0	43.4	40.7	41.9	45.1
Diabetes duration (years)	8 (3–15)	7 (2–15)	10 (3–13.5)	8 (2–15)	7.5 (3–14)
Dyslipidemia,^a^ %	94.3	94.1	94.9	93.5	95.2
Statin use, %	77.8	78.6	72.9	79.1	73.2
Arterial hypertension, %	84.8	84.8	84.7	81.5	88.9
Antihypertensive treatment					
ACEi/ARBs, %	79.8	79.1	84.2	78.3	82.0
Diuretics, %	59.1	57.8	66.7	57.5	62.0
Calcium channel blockers, %	28.3	28.3	28.1	24.8	33.3
Systolic BP (mmHg)	138 (17)	138 (17)	137 (17)	138 (18)	138 (16)
Diastolic BP (mmHg)	77 (10)	77 (10)	78 (10)	77 (10)	78 (10)
Cardiovascular diseases, %	26.8	26.4	28.8	25.6	27.5
Microvascular complications, %	49.0	48.3	52.7	49.1	48.3
Diabetes treatment, %					
Metformin	89.9	89.1	94.7	87.6	92.7
Sulfonylureas	43.9	43.4	47.4	45.1	42.0
Insulin	46.3	45.7	49.2	44.2	49.0
Other antidiabetic drugs^b^	6.2	5.3	10.2	5.9	6.8
Aspirin	88.0	88.3	86.4	85.5	90.8
Laboratory parameters					
Fasting glucose (mmol/l)	7.8 (3.1)	7.8 (3.0)	8.2 (3.7)	7.6 (3.1)	8.2 (3.1)
HbA_1c_ (%)	7.8 (1.5)	7.8 (1.5)	7.8 (1.6)	7.6 (1.5)	8.0 (1.5)‡
(mmol/mol)	61.7 (10.5)	61.7 (10.5)	61.7 (11.6)	59.6 (10.5)	63.9 (10.5)‡
Triacylglycerol (mmol/l)	1.4 (1.0–2.0)	1.4 (1.1–1.9)	1.5 (1.0–2.3)	1.3 (0.9–1.7)	1.7 (1.2–2.3)*
HDL-cholesterol (mmol/l)	1.2 (0.3)	1.2 (0.3)	1.1 (0.3)‡	1.2 (0.3)	1.1 (0.3)*
LDL-cholesterol (mmol/l)	2.4 (0.8)	2.5 (0.8)	2.1 (0.8)†	2.4 (0.8)	2.4 (0.8)
TE parameters					
LSM (kPa)	7.3 (5.9)	5.6 (1.8)	17.3 (10.2)*	6.6 (6.2)	8.5 (5.4)*
CAP (dB/m)	279 (59)	274 (59)	306 (55)*	241 (41)	336 (27) *
Outcomes incidence, absolute number (incidence rate per 1000 person-years of follow-up)					
Total CVEs	69 (32.7)	53 (29.4)	16 (52.1)‡	47 (40.8)	22 (26.3)
MACEs	50 (23.6)	37 (20.4)	13 (42.3)‡	35 (30.1)	15 (18.0)
All-cause mortality	85 (39.5)	70 (38.2)	15 (46.8)	60 (51.2)	25 (29.2)†
CV mortality	40 (18.6)	31 (16.9)	8 (25.0)	30 (25.6)	10 (11.7)‡
Non-CV mortality	45 (20.9)	38 (20.7)	7 (21.9)	30 (25.6)	15 (17.5)

*LSM* liver stiffness measurement, *CAP* controlled attenuation parameter, *BMI* body mass index, *ACEi* angiotensin-converting enzyme inhibitor, *ARBs* angiotensin II receptor blockers, *HbA1c* glycated hemoglobin, *CVEs* cardiovascular events, *MACEs* major adverse cardiovascular events, *CV* cardiovascular

^a^Dyslipidemia was defined by a serum total cholesterol > 5.2 mmol/l, or LDL-cholesterol > 3.4 mmol/l, or HDL-cholesterol < 1.3 mmol/l in women or < 1.0 mmol/l in men, or triglycerides > 1.7 mmol/l, or by using any lipid-lowering medication

^b^25 patients were using other antidiabetic medications: 17 were using DPP-4 inhibitors and 10 were using thiazolidinediones (2 were using both), none were using GLP-1 agonists or SGLT-2 inhibitors

Values are means (SDs) or proportions, except for diabetes duration and serum triacylglycerol, which are medians (interquartile range). *p < 0.001; †p < 0.01; ‡p < 0.05; for bivariate comparisons between subgroups with higher and lower LSM and CAP

### Follow-up and outcomes incidences

**Fig. 1:**
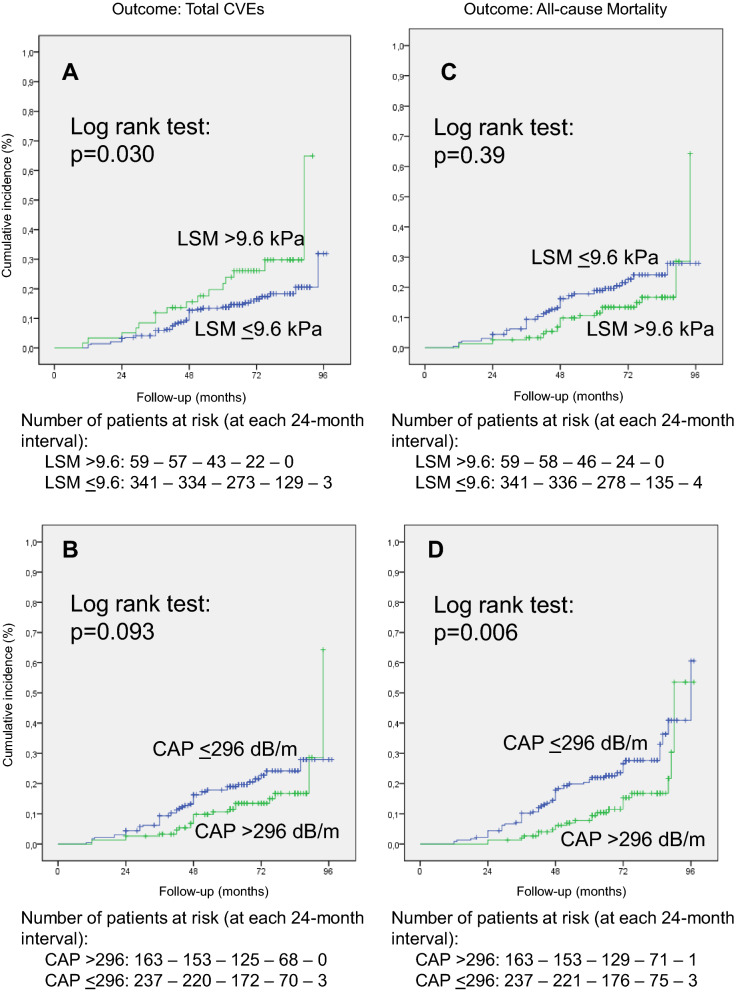
Kaplan-Meier curves of cumulative incidence of total cardiovascular events (CVEs, left panels **A** and **B**) and of all-cause mortality (right panels **C** and **D**) in 400 patients with nonalcoholic fatty liver disease and type 2 diabetes divided according to having or not advanced liver fibrosis (liver stiffness measurement [LSM] > 9.6 kPa, top panels **A** and **C**) and severe steatosis (controlled attenuation parameter [CAP] > 296 dB/m, bottom panels **B** and **D**) on hepatic transient elastography

During a median follow-up after TE examination of 5.5 years (maximum 8 years), which corresponded to 2188 person-years (PY) of follow-up, there were 69 total CVEs (incidence rate: 32.7 per 1000 PY), 50 MACEs (23.6 per 1000 PY), and 85 all-cause deaths (39.5 per 1,000 PY), 40 from cardiovascular causes (18.6 per 1000 PY) and 45 from non-cardiovascular causes (20.9 per 1000 PY). The main causes of non-cardiovascular deaths were infections (20 cases), cancer (14 cases, 3 hepatocarcinomas) and renal failure (6 cases). Table 1 (bottom) outlines the absolute numbers and incidence rates of each adverse outcome in participants with and without advanced fibrosis and severe steatosis. Patients with advanced fibrosis had higher incidences of cardiovascular and mortality outcomes than those without advanced fibrosis (with significant differences for total CVEs and MACEs incidences); whereas patients with severe steatosis had lower incidences of these outcomes than those without severe steatosis (with significant differences for all-cause and cardiovascular mortality incidences). Kaplan-Meier estimation of cumulative incidences of total CVEs and all-cause mortality, shown on Fig. 1, confirmed these observations.

### Risks associated with increasing LSM and CAP and improvements in risk discrimination

Table 2 presents the adjusted risks associated with increasing LSM and CAP, both separately and concomitantly included into the same model. When analyzed as continuous variables, an increasing LSM was a significant risk marker for total CVEs, MACEs, all-cause and non-cardiovascular mortality, with adjusted HRs varying from 1.04 to 1.05, estimated for increments of 1 kPa in LSM; whereas an increasing CAP was a significant protective factor for total CVEs, MACEs, all-cause and cardiovascular mortality, with HRs varying from 0.90 to 0.93, estimated for increments of 10 dB/m en CAP. When analyzed as dichotomical variables, a high LSM (> 9.6 kPa) was a significant risk marker for total CVEs, MACEs and cardiovascular mortality, with adjusted HRs ranging from 2.5 to 3.0; whereas a high CAP (> 296 dB/m) was a significant protective factor for total CVEs, MACEs, all-cause and cardiovascular mortality, with adjusted HRs from 0.3 to 0.5. In general, when included simultaneously, LSM and CAP were additive in their predictive performance. Dichotomizing CAP at higher values (> 330 dB/m) did not materially change any of the results (Table 2). Also, further adjustment for sulphonylureas use did not change the results. In interaction and sensitivity analyses (shown in Table 3) there were no significant interactions between LSM or CAP with any of the selected clinical parameters, except between CAP and smoking status for all-cause mortality, where the protective effect of increasing CAP was more marked in current or past smokers than in non-smokers. When cross-classified into 4 subgroups according to high/low LSM and CAP, the subgroup with the lowest risks of adverse outcomes was that with low LSM/high CAP and the subgroup with highest risks was the opposite subgroup (high LSM/low CAP), with reciprocally-adjusted HRs of 0.13 (95% CI: 0.06–0.30) and 7.70 (95% CI: 3.38–17.55) for total CVEs (p < 0.001); and 0.25 (95% CI: 0.10–9.61) and 3.95 (95% CI: 1.62–9.61) for all-cause mortality (p = 0.003), respectively. The other 2 subgroups (low LSM/low CAP and high LSM/high CAP) had non-significant excess risks in relation to these two extreme risks subgroups.

**Table 2:** Risks of adverse outcomes associated with higher liver stiffness measurement (LSM, indicating fibrosis) and controlled attenuation parameter (CAP, indicating liver steatosis) on hepatic transient elastography (TE) in 400 patients with type 2 diabetes and NAFLD

Outcomes	Model 1^a^		Model 2^b^	
TE parameters	HR (95% CI)	p-value	HR (95% CI)	p-value
Total Cardiovascular Events (n = 69)				
LSM (1 kPa increase)	1.04 (1.00-1.08)	0.037	1.05 (1.01–1.08)	0.007
CAP (10 dB/m increase)	0.94 (0.90–0.99)	0.022	0.93 (0.89–0.98)	0.008
LSM > 9.6 kPa	1.99 (1.09–3.62)	0.024	2.66 (1.41–5.02)	0.002
CAP > 296 dB/m	0.53 (0.31–0.92)	0.023	0.44 (0.25–0.78)	0.005
CAP > 330 dB/m	0.42 (0.19–0.93)	0.032	0.36 (0.16–0.80)	0.012
Major Adverse Cardiovascular Events (n = 50)				
LSM (1 kPa increase)	1.05 (1.00–1.09)	0.041	1.05 (1.01–1.09)	0.011
CAP (10 dB/m increase)	0.92 (0.87–0.98)	0.006	0.91 (0.86–0.97)	0.002
LSM > 9.6 kPa	2.26 (1.14–4.49)	0.020	3.03 (1.47–6.26)	0.003
CAP > 296 dB/m	0.45 (0.24–0.87)	0.018	0.37 (0.19–0.73)	0.004
CAP > 330 dB/m	0.34 (0.13–0.91)	0.031	0.29 (0.11–0.78)	0.014
All-cause Mortality (n = 85)				
LSM (1 kPa increase)	1.04 (1.01–1.07)	0.012	1.04 (1.01–1.07)	0.004
CAP (10 dB/m increase)	0.93 (0.89–0.97)	0.002	0.92 (0.88–0.97)	0.001
LSM > 9.6 kPa	1.58 (0.87–2.87)	0.13	1.70 (0.90–3.21)	0.10
CAP > 296 dB/m	0.53 (0.31–0.89)	0.016	0.50 (0.29–0.85)	0.010
CAP > 330 dB/m	0.53 (0.26–1.07)	0.077	0.50 (0.25–1.02)	0.057
Cardiovascular Mortality (n = 40)				
LSM (1 kPa increase)	1.02 (0.96–1.08)	0.47	1.04 (0.99–1.10)	0.14
CAP (10 dB/m increase)	0.91 (0.85–0.97)	0.006	0.90 (0.84–0.97)	0.003
LSM > 9.6 kPa	1.68 (0.73–3.84)	0.22	2.46 (1.02–5.95)	0.045
CAP > 296 dB/m	0.38 (0.18–0.83)	0.015	0.32 (0.14–0.71)	0.005
CAP > 330 dB/m	0.35 (0.12–1.04)	0.058	0.30 (0.10–0.92)	0.035
Non-Cardiovascular Mortality (n = 45)				
LSM (1 kPa increase)	1.05 (1.01–1.09)	0.010	1.05 (1.01–1.09)	0.014
CAP (10 dB/m increase)	0.94 (0.88–1.01)	0.073	0.94 (0.88-1.00)	0.066
LSM > 9.6 kPa	1.34 (0.55–3.24)	0.52	1.09 (0.41–2.91)	0.86
CAP > 296 dB/m	0.68 (0.33–1.39)	0.29	0.68 (0.33–1.39)	0.29
CAP > 330 dB/m	0.76 (0.29–1.98)	0.58	0.76 (0.30–1.98)	0.58

Values are hazard ratios (HR) with 95% confidence intervals

*TE* transient elastography, *LSM* liver stiffness measurement, *CAP* controlled attenuation parameter

^a^Model 1 was adjusted for age, sex, diabetes duration, BMI, smoking, arterial hypertension, presence of atherosclerotic cardiovascular diseases and microvascular complications at baseline, serum HbA1c, LDL- and HDL-cholesterol, and use of insulin, statins and aspirin

^b^Model 2 was further concomitantly adjusted for both LSM and CAP

**Table 3:** Sensitivity and interaction analyses between liver transient elastography parameters and selected clinical characteristics for total cardiovascular events and all-cause mortality outcomes

	Total Cardiovascular Events (n = 69)				All-cause Mortality (n = 85)			
	LSM		CAP		LSM		CAP	
Characteristic	HR (95 %CI)	P_interaction_	HR (95 %CI)	P_interaction_	HR (95 %CI)	P_interaction_	HR (95 %CI)	P_interaction_
Age < 65 years (n = 191)	1.00 (0.94–1.07)	0.85	0.91 (0.84–0.99)*	0.78	1.01 (0.95–1.07)	0.59	0.88 (0.80–0.96)*	0.71
≥65 years (n = 209)	1.05 (1.00–1.10)*		0.97 (0.90–1.04)		1.04 (1.00–1.08)		0.95 (0.89–1.00)	
Female sex (n = 236)	1.04 (0.99–1.09)	0.60	0.96 (0.90–1.02)	0.18	1.04 (0.99–1.09)	0.83	0.89 (0.84–0.95)*	0.20
Male sex (n = 144)	1.03 (0.98–1.09)		0.95 (0.87–1.03)		1.04 (1.00–1.07)		0.98 (0.91–1.06)	
Non-obese (n = 198)	1.05 (0.98–1.11)	0.92	0.96 (0.89–1.03)	0.66	1.05 (1.01–1.09)*	0.52	0.95 (0.89–1.01)	0.56
Obese (n = 202)	1.04 (0.99–1.08)		0.93 (0.86-1.00)*		1.03 (0.98–1.07)		0.89 (0.83–0.96)*	
Never smokers (n = 228)	1.01 (0.94–1.08)	0.21	0.99 (0.92–1.06)	0.20	1.02 (0.95–1.08)	0.33	0.98 (0.92–1.04)	0.044
Current/past smokers (n = 172)	1.05 (1.01–1.09)*		0.87 (0.80–0.95)*		1.04 (1.01–1.07)*		0.88 (0.82–0.94)*	
Without CVD (n = 293)	1.04 (0.98–1.09)	0.72	0.94 (0.88–1.01)	0.88	1.04 (1.00-1.08)*	0.80	0.91 (0.86–0.97)*	0.14
With CVD (n = 107)	1.05 (0.99–1.11)		0.93 (0.86-1.00)		1.03 (0.96–1.09)		0.92 (0.86-1.00)*	
Without microvasc (n = 204)	1.04 (0.98–1.10)	0.72	0.91 (0.84–0.99)*	0.77	1.04 (1.01–1.08)*	0.34	0.89 (0.83–0.97)*	0.10
With microvasc (n = 196)	1.02 (0.96–1.09)		0.96 (0.89–1.03)		1.02 (0.97–1.08)		0.94 (0.88–1.00)	

Values are hazard ratios (95% confidence intervals) estimated for increments of 1 kPa in LSM and 10 dB/m in CAP, and adjusted for the same covariates as in model 1 from Table 2, except the respective stratifying variable

*LSM* liver stiffness measurement, *CAP* controlled attenuation parameter, *HR* hazard ratio, *CI* confidence interval, * microvasc* microvascular complications

*p < 0.05

Table 4 presents the improvement in risk discrimination after adding TE parameters over a standard risk factor model for total CVEs and all-cause mortality, as evaluated by increase in C-statistics and relative IDI index. There were significant improvements in discrimination after adding LSM for all-cause mortality risk and after adding CAP to total CVEs and mortality risk. Adding both CAP and LSM provided further risk discrimination improvements for both outcomes.

**Table 4:** Improvements in risk discrimination of adverse outcomes after adding LSM, CAP or both to a basic risk factor model

Predictive models	Total CVEs (n = 69)		All-cause mortality (n = 85)	
	C-statistic AUC (95% CI)	Relative IDI (%)	C-statistic AUC (95% CI)	Relative IDI (%)
Basic model^a^	0.745 (0.681–0.810)	–	0.738 (0.676–0.800)	–
Adding LSM	0.756 (0.693–0.819)	7.8 %	0.751 (0.691–0.812)	14.1%*
Adding CAP	0.764 (0.701–0.827)*	22.7%*	0.763 (0.706–0.820)*	37.2%*
Adding LSM and CAP	0.777 (0.717–0.838)*	33.6%*	0.775 (0.719–0.832)*	52.1%*

Values are area under curves (95% confidence intervals) for C-statistic and relative percentage increase in Integrated Discrimination Improvement (IDI) index

*CVEs* cardiovascular events, *AUC* area under curve, *CI* confidence interval, *IDI* Integrated Discrimination Improvement, *LSM* liver stiffness measurement, *CAP* controlled attenuation parameter

^a^The basic risk factor model included age, sex, diabetes duration, BMI, smoking, arterial hypertension, presence of atherosclerotic cardiovascular diseases and microvascular complications at baseline, serum HbA1c, LDL- and HDL-cholesterol, and use of insulin, statins and aspirin

*Significant (p < 0.05) increases in relation to the basic model

## Discussion

This longitudinal prospective study with 400 individuals with NAFLD and type 2 diabetes followed-up for a median of 5.5 years after baseline TE has three main findings. First, it showed that LSM, both as a continuous variable and categorized at > 9.6 kPa (which reflects advanced liver fibrosis), was associated with higher risks of total CVEs and MACEs, and of all-cause mortality. Second, both increasing values of CAP and severe steatosis (defined by a CAP > 296 dB/m or > 330 dB/m) were protective for total CVEs, MACEs and for all-cause and cardiovascular mortality. Third, LSM and CAP were able to improve risk discrimination for adverse cardiovascular outcomes and mortality. Overall, liver fibrosis assessed by LSM was associated with an increased cardiovascular and mortality risk, whereas liver steatosis assessed by CAP conferred a protective effect for these outcomes; and these effects were independent and additive over each one. Hence, we demonstrated that both parameters obtained by TE may be useful in risk stratification of individuals with NAFLD and type 2 diabetes.

Although TE has been well-validated to assess the extent of liver fibrosis and steatosis in NAFLD [12–16], data about LSM and CAP for prediction of events in NAFLD are scarce [17–20]. In a retrospective analysis of consecutive individuals with NAFLD, with a median follow-up of 35 months, LSM at baseline and its changes during follow-up predicted liver-related outcomes and all-cause mortality [17]. In another retrospective study including suspected NAFLD, hepatitis B and C, and other causes of hepatic disease, LSM was one the predictors of liver-related outcomes; however, in the analysis of the NAFLD subgroup, neither the presence nor the severity of hepatic steatosis measured by CAP predicted liver-related outcomes, cancer, or CVEs after a median follow-up of 26 months [18]. A prospective investigation in NAFLD patients followed-up for a median of 27 months demonstrated that LSM was a predictor of all-cause mortality [19]. One previous study with 529 patients with type 2 diabetes [29] suggested that the presence of NAFLD, defined by CT imaging (liver/spleen attenuation rate, which detects liver steatosis, not fibrosis) was predictive of cardiovascular events occurrence over a median follow-up of 4.4 years. Another study with 1120 type 2 diabetic patients [30] reported that only the subgroup with liver fibrosis, defined by the FIB-4, a non-invasive laboratorial score, had progressive carotid intima-media thickening over a median follow-up of 6–8 years. However, only one previous study investigated LSM and CAP usefulness in risk stratification for cardiovascular morbidity and mortality and all-cause mortality in 454 individuals with type 2 diabetes [20]. Nonetheless, this study had several limitations that precluded a comprehensive analysis. The median follow-up was only 2 years, and the primary outcome, all-cause mortality, occurred only in 3.7% of the individuals; and the secondary outcome, which occurred in 23%, included a composite of several non liver-related morbidity outcomes. Different from our findings, they did not find any associations between TE parameters and the outcomes [20]. However, similar to our findings, in this study the subgroup with more severe steatosis had the lowest incidence of adverse outcomes [20]. In the present study, we followed the patients for a longer time than these studies (median of 5.5 years), and we had a greater number of events than the studies from Liu et al. [18] and Grgurevic et al. [20], which allowed us to analyze separately total CVEs, MACEs, and cardiovascular and all-cause mortality. We demonstrated that LSM was a predictor of total CVEs, MACEs, and all-cause mortality. When categorized at 9.6 kPa, it also predicted cardiovascular mortality. In a systematic review and meta-analysis, biopsy-confirmed fibrosis was associated with risk of mortality and liver-related morbidity in patients with NAFLD, with and without adjustment for confounding factors [10]. In another cohort study with a very long follow-up (33 years), advanced histological NAFLD fibrosis stages were predictors of all-cause and disease-specific mortality, including CVD, while inflammatory activity grade was not associated with mortality risk [31]. The pathophysiological mechanisms behind the association of liver fibrosis in NAFLD with cardiovascular disease are still incompletely understood and may involve other pathways besides insulin resistance, such as oxidative stress, inflammation, and gut microbiota [32]. Another possible physiopathological link between advanced liver fibrosis and atherosclerotic disease development in diabetes was the finding that individuals with NAFLD and diabetes who increased or persisted with abnormally high aortic stiffness during follow-up were also at particular risk of having advanced liver fibrosis [23]. Hence, increased aortic stiffness, a well-established risk factor for adverse cardiovascular outcomes [33], may be mediating, at least partially, the association between advanced liver fibrosis and CVD risk in diabetic patients.

The finding that liver steatosis assessed by CAP may be protective for cardiovascular events and cardiovascular mortality, either as a continuous variable or categorized as severe steatosis, is new and but not completely unexpected. Based on observational studies that showed a relation between steatosis/NAFLD and an increased risk of coronary artery disease, it was thought that the whole spectrum of NAFLD would be associated with increased risk of coronary artery disease. However, a Mendelian randomization study exploring the epidemiological association between NAFLD and cardiovascular diseases carried out in two large European cohorts found no evidence for a causal relationship between NAFLD and cardiovascular disease by examining two steatogenic single nucleotide polymorphisms (SNPs) in the patatin-like phospholipase domain-containing 3 (PNPLA3) gene and in the transmembrane 6 superfamily member 2 (TM6SF2) gene [34].The findings persisted when meta-analysed together with the CARDIoGRAMplus C4D consortium data [34, 35]. Indeed, the odds ratios of the associations between severe liver steatosis induced by these two SNPs with incident coronary artery disease were lower than 1 in all analyses, either in analysis of each separate cohort or when meta-analyzed together, suggesting a possible protective effect of liver steatosis for CVD development [34]. Specifically the TM6SF2 gene polymorphism is also associated with lower serum LDL-cholesterol and triglyceride levels, which may help to explain its protective cardiovascular effect [36]; however, this does not occur with the PNPLA3 variant [34]. Other large genetic study had already demonstrated that these two SNPs were associated with higher liver fat but lower coronary artery disease risk [37]. Hence, our findings support these reports, by showing that the entire spectrum of NAFLD might have dual opposing effects on cardiovascular and mortality risk, being protective for higher steatosis and hazardous for advanced fibrosis, at least in individuals with type 2 diabetes. We may speculate that severe liver steatosis *per se* may have cardiovascular protective effects, which might be driven at least in part by these steatogenic genetic polymorphisms. The NAFLD phenotype with high steatosis and low fibrosis, which might be an earlier stage, is the best one in terms of risk of adverse prognosis in patients with diabetes. However, patients may evolve to different phenotypes of the NAFLD spectrum: they may persist with marked steatosis and develop fibrosis, which did not appear to increase their risks of adverse outcomes; on the other hand, they may reduce liver fat accumulation and develop fibrosis, which seemed to greatly increase their cardiovascular and mortality risks. Clearly, these are speculations because we did not evaluate physiopathological mechanisms, neither performed serial TE examinations to evaluate transitions in liver steatosis and fibrosis over time.

This study has some limitations to notice. First, we did not have liver biopsy to correlate with findings of TE, but this method has a reasonable accuracy for estimating liver fibrosis and steatosis [12–16]. Indeed, there is no non-invasive gold standard method for evaluating liver fibrosis; and the gold standard method, liver biopsy, for many reasons is not ideal for serial evaluation of hepatic disease. Second, although we had a relatively large cohort of patients with diabetes and NAFLD followed-up for a long period of time, we still had few outcomes, particularly the separated cardiovascular and non-cardiovascular deaths and also for the subgroup analyses. Hence, these specific analyses should be faced with caution and the findings might be considered as exploratory and hypothesis-generating. Third, we did not have serial TE measurements, so changes in LSM or in CAP during follow-up could not be evaluated. Fourth, establishing a cut-off for CAP might raise some concern since the last European Association for the Study of the Liver (EASL) guideline for non-invasive tests highlights the limitations of CAP to quantify steatosis [38]. Otherwise, this guideline recognizes that CAP is a promising technique to detect steatosis, and it recommends a cut-off of 275 dB/m for diagnosing steatosis. Hence, our cut-offs of 296 and 330 dB/m probably included patients with progressively greater degree of steatosis. Fifth, although we adjusted our analyses to the classic cardiovascular risk factors, as in all observational cohort studies, some residual confounding due to unmeasured factors might still exist. Finally, our findings are applicable to middle-aged to elderly NAFLD individuals with diabetes followed at a tertiary-care center, and may not be generalized to younger individuals followed at primary care or to non-diabetic individuals with NAFLD. On the other hand, this study’s main strength is its well-documented cohort followed-up regularly with standardized care and annual outcomes evaluation, which permitted a comprehensive analysis of the associations between TE parameters and cardiovascular and mortality outcomes.

## Conclusions

We demonstrated in a cohort of individuals with NAFLD and type 2 diabetes that CAP and LSM, evaluated by TE, are useful for risk stratification of adverse cardiovascular outcomes and mortality. These two TE parameters have dual opposing and additive effects: CAP, reflecting liver steatosis, is protective, whereas LSM, reflecting liver fibrosis, is hazardous. Other larger longitudinal studies exploring their predictive roles in individuals with NAFLD and diabetes are necessary. If confirmed, then future interventional studies aiming to reduce liver fibrosis preferably without affecting steatosis may be designed to evaluate whether it can improve cardiovascular and mortality prognoses in these high-risk individuals with NAFLD and diabetes.

## Data Availability

The Rio de Janeiro Type 2 Diabetes Cohort Study is an on-going study, and its dataset is not publicly available due to individual privacy of the participants. However, it may be available from the corresponding author on reasonable request.

## Abbreviations

NAFLD: Nonalcoholic fatty liver disease
TE: Transient elastography
LSM: Liver stiffness measurement
CAP: Controlled attenuation parameter
BMI: Body mass index
HbA1c: Glycated hemoglobin
CVEs: Cardiovascular events
MACEs: Major adverse cardiovascular events
CV: Cardiovascular
